# Ethnic differences in stroke outcomes in Aotearoa New Zealand: A national linkage study

**DOI:** 10.1177/17474930231164024

**Published:** 2023-03-24

**Authors:** Hayley J Denison, Marine Corbin, Jeroen Douwes, Stephanie G Thompson, Matire Harwood, Alan Davis, John N Fink, P Alan Barber, John H Gommans, Dominique A Cadilhac, William Levack, Harry McNaughton, Joosup Kim, Valery L Feigin, Virginia Abernethy, Jackie Girvan, Andrew Wilson, Anna Ranta

**Affiliations:** 1 Research Centre for Hauora and Health, Massey University, Wellington, New Zealand; 2 Department of Medicine, University of Otago Wellington, Wellington, New Zealand; 3 Department of Medicine, University of Auckland, Auckland, New Zealand; 4 Whangarei Hospital, Whangarei, New Zealand; 5 Christchurch Hospital, Christchurch, New Zealand; 6 Hawke’s Bay Hospital, Hastings, New Zealand; 7 Department of Medicine, School of Clinical Sciences, Monash University, Clayton, VIC, Australia; 8 Medical Research Institute of New Zealand, Wellington, New Zealand; 9 Auckland University of Technology, Auckland, New Zealand; 10 Stroke Foundation New Zealand, Wellington, New Zealand; 11 Independent Consumer, Ashburton, New Zealand; 12 Wairau Hospital, Blenheim, New Zealand

**Keywords:** Disparities, ethnicity, indigenous, stroke, data linkage

## Abstract

**Background:**

Ethnic differences in post-stroke outcomes have been largely attributed to biological and socioeconomic characteristics resulting in differential risk factor profiles and stroke subtypes, but evidence is mixed.

**Aims:**

This study assessed ethnic differences in stroke outcome and service access in New Zealand (NZ) and explored underlying causes in addition to traditional risk factors.

**Methods:**

This national cohort study used routinely collected health and social data to compare post-stroke outcomes between NZ Europeans, Māori, Pacific Peoples, and Asians, adjusting for differences in baseline characteristics, socioeconomic deprivation, and stroke characteristics. First and principal stroke public hospital admissions during November 2017 to October 2018 were included (N = 6879). Post-stroke unfavorable outcome was defined as being dead, changing residence, or becoming unemployed.

**Results:**

In total, 5394 NZ Europeans, 762 Māori, 369 Pacific Peoples, and 354 Asians experienced a stroke during the study period. Median age was 65 years for Māori and Pacific Peoples, and 71 and 79 years for Asians and NZ Europeans, respectively. Compared with NZ Europeans, Māori were more likely to have an unfavorable outcome at all three time-points (odds ratio (OR) = 1.6 (95% confidence interval (CI) = 1.3–1.9); 1.4 (1.2–1.7); 1.4 (1.2–1.7), respectively). Māori had increased odds of death at all time-points (1.7 (1.3–2.1); 1.5 (1.2–1.9); 1.7 (1.3–2.1)), change in residence at 3 and 6 months (1.6 (1.3–2.1); 1.3 (1.1–1.7)), and unemployment at 6 and 12 months (1.5 (1.1–2.1); 1.5 (1.1–2.1)). There was evidence of differences in post-stroke secondary prevention medication by ethnicity.

**Conclusion:**

We found ethnic disparities in care and outcomes following stroke which were independent of traditional risk factors, suggesting they may be attributable to stroke service delivery rather than patient factors.

## Introduction

Stroke is a leading cause of death and disability.^1^ Best-practice stroke care promotes favorable outcomes, but evidence suggests that significant inequity in access exists for ethnic minorities, potentially resulting in poorer stroke outcomes.^2,3^ For example, a US registry–based study showed that access to evidence-based treatments was lower among African Americans compared with Americans of European descent, even when managed in the same service.^2^

Evidence from research on post-stroke outcomes for different ethnicities has been mixed and has largely focused on mortality.^4^ Some studies have found that stroke mortality is higher for indigenous and other ethnic minority groups.^5,6^ However, other studies reported no differences^7^ or found lower post-stroke mortality for ethnic minorities.^8^ Studies that focused on functional improvement^9^ and independence,^10^ physical functioning,^11^ and employment status^12^ also showed mixed results.

This study aimed to identify the factors that contribute to improved outcomes for ethnic minorities in New Zealand (NZ) by assessing differences in mortality, functional independence, and access to appropriate stroke management, while controlling for patient characteristics and stroke severity.

## Methods

### Study design

This whole-population study used routinely collected linked data to compare post-stroke outcomes between ethnic groups and is part of a larger research program^13^ that will inform the NZ stroke strategy and may guide stroke services internationally. Approval was obtained from the Central Region Health and Disability Ethics Committee (17CEN164).^13^

### Data source

Data were obtained from Stats NZ’s Integrated Data Infrastructure (IDI), a database of de-identified administrative and survey data about people and households in NZ.^14^ It includes data about health, education, income, social support payments, migration, and other life events, which can be linked at the individual level. The IDI provides a longitudinal record of events.

### Study cohort

A cohort of people aged ⩾16 years who had a stroke between 1 November 2017 and 31 October 2018 was established for the whole NZ population. All public hospital admissions with stroke International Classification of Diseases (ICD) codes (I61 = intracerebral hemorrhage, I63 = cerebral infarction, I64 = stroke unspecified) as a principal diagnosis during this period were selected for the 2017 resident population. Where someone had >1 stroke during this period, the first was used.

### Ethnicity

Self-identified ethnicity was obtained from public hospital data and grouped into NZ European, Māori, Pacific Peoples, Asian, and Other for whom data were not reported given low stroke numbers (n = 30).

### Primary outcome: stroke unfavorable outcome

As modified Rankin Scale data were not available, a composite unfavorable outcome of death (birth, death, and marriage data), address change (address notification data), or job loss (Inland Revenue Department’s tax data) was defined at 3-, 6-, and 12-months post-stroke. People not working at the time of stroke were assigned a favorable status for the unemployed outcome. We used change in residence as a surrogate to indicate discharge to either residential care or moving in with family, both indicating post-stroke disability warranting additional support with daily activities. Analyses were conducted for the unfavorable outcome as well as for each of the three components. Sensitivity analyses excluding people not working at the time of stroke or with a prior stroke were conducted, as well as stratified analyses by stroke subtype (I61/I63/I64).

### Other outcomes

Secondary outcomes included performance of thrombectomy (hospital discharge data) and medication dispensing after stroke (pharmaceutical data).

### Statistical analyses

Analyses were conducted using SAS Enterprise Guide version 7.1. Logistic regression was used to estimate odds ratios (ORs) for associations between ethnicity and outcomes, with NZ Europeans as the reference group. Analyses were adjusted for age, sex, socioeconomic deprivation (1–2 (least); 3–4; 5–6; 7–8; 9–10 (most)) using the NZ Deprivation Index 2013 (a census-based index with a relative deprivation score based on place of residence),^15^ location of hospital (urban/non-urban), stroke type (intracerebral hemorrhage, cerebral infarction, or unspecified stroke), hypertension, dyslipidemia, atrial fibrillation, and length of stay (indicator of stroke severity).^16^ Hospitals were categorized as urban if they were within a 25-km radius of an urban center with a population of >100,000. Supplemental Table S1 shows the definitions of co-morbidities.

Smoking data were collected from the 2018 Census. Due to a large number of missing data (n = 753, 10.9%) and results being similar after adjustment, we excluded smoking in final regression models.

### Reporting

The IDI confidentiality requirements necessitate that counts are randomly rounded up or down to the next multiple of 3 and percentages calculated from the rounded counts. Therefore, the total numbers in each figure vary slightly and may not add to 100%. However, statistical tests were performed on the unrounded counts. Counts under six and the results of the associated tests were suppressed and marked as “S” in the table/figures.

## Results

The resident population in 2017 consisted of 4,743,345 people. During the study period, 6879 people meeting inclusion criteria had a primary diagnosis of incident stroke (a typical annual incidence for NZ). The age-standardized incidence was 93.5/100,000 for NZ Europeans, 139.2/100,000 for Māori, 173.1/100,000 for Pacific Peoples, and 64.0/100,000 for Asians and Other.

Median (interquartile range) age for Māori, Pacific Peoples, Asians, and NZ Europeans was 65 (20), 65 (22), 71 (19), and 79 (16), respectively (Table 1). Ever smoking was the highest among Māori (55%), followed by NZ Europeans (40%), Pacific Peoples (28%), and Asians (16%). Over 50% of Māori and Pacific Peoples were in the most socioeconomically deprived quintile compared with <25% among NZ Europeans and Asians. More Pacific Peoples (91%) and Asians (93%) were admitted to urban hospitals than NZ Europeans (61%) and Māori (51%).

**Table 1.** Demographics and baseline characteristics of stroke patients by ethnicity.

	NZ European		Māori		Pacific Peoples		Asians	
	n	%	n	%	n	%	n	%
Total	5394		762		369		354	
Females	2592	48.05	420	55.12	186	50.41	168	47.46
Age (median, interquartile range)	79.1 (16.1)		64.7 (19.8)		64.9 (22.2)		70.7 (19.1)	
Ever smoked (n missing = 753)	2175	40.32	417	54.72	105	28.46	57	16.10
NZDep^a^ (n missing = S)								
1–2 (least deprived)	861	15.96	36	4.72	18	4.88	45	12.71
3–4	1116	20.69	51	6.69	21	5.69	69	19.49
5–6	1212	22.47	84	11.02	42	11.38	69	19.49
7–8	1278	23.69	183	24.02	63	17.07	84	23.73
9–10 (most deprived)	924	17.13	411	53.94	228	61.79	84	23.73
Hypertension^b^	4341	80.48	597	78.35	312	84.55	258	72.88
Dyslipidemia^c^	3285	60.90	468	61.42	249	67.48	192	54.24
Diabetes	1338	24.81	309	40.55	207	56.10	153	43.22
Atrial fibrillation^d^	1722	31.92	285	37.40	93	25.20	54	15.25
Prior stroke	936	17.35	123	16.14	69	18.70	57	16.10
Prior transient ischemic attack	549	10.18	66	8.66	21	5.69	18	5.08
Prior myocardial infarction	726	13.46	93	12.20	42	11.38	36	10.17
Urban hospital (n missing = 93)	3306	61.29	387	50.79	336	91.06	330	93.22
Length of stay in days (median, interquartile range)	4 (5)		4 (5)		5 (5)		5 (6)	
Stroke type								
Intracerebral hemorrhage (161)	645	11.96	57	7.48	66	17.89	90	25.42
Ischemic stroke/cerebral infarction (163)	4422	81.98	645	84.65	291	78.86	255	72.03
Stroke otherwise unspecified (164)	324	6.01	60	7.87	15	4.07	9	2.54

a New Zealand Deprivation Index.

b Two or more antihypertensive prescriptions in the 10 years prior to stroke event or public hospital discharge for hypertension (see Supplemental Table S1 in Supplemental Material for detailed frequencies).

c Two or more prescriptions used to lower cholesterol in the 10 years prior to stroke event or public hospital discharge for dyslipidemia (see Supplemental Table S1 for detailed frequencies).

d Two or more anticoagulant prescriptions in the 10 years prior to stroke event or public hospital discharge for atrial fibrillation (see Supplemental Table S1 for detailed frequencies).

Pacific Peoples were most affected by hypertension (85%), diabetes (56%), and dyslipidemia (67%). Over 30% of NZ Europeans and Māori had atrial fibrillation compared with Pacific Peoples (25%) and Asians (15%) (Table 1).

### Unfavorable outcomes

**Figure 1.**
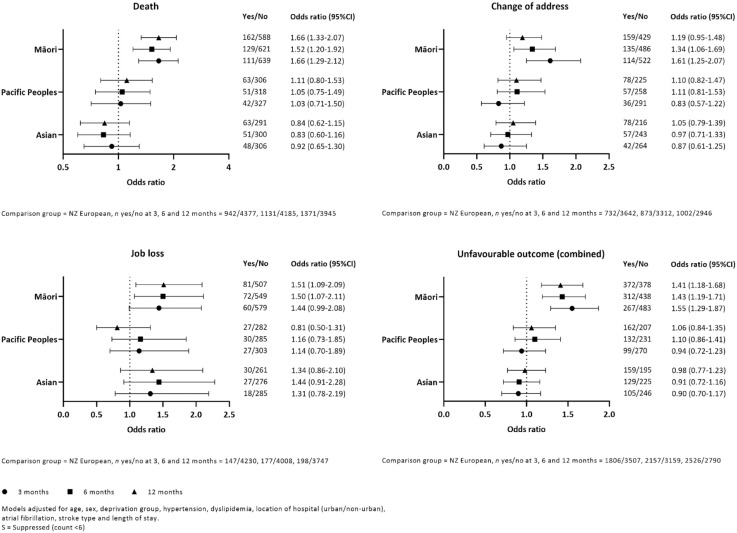
Risks of individual and combined unfavorable outcomes by ethnicity.

Compared with NZ Europeans, Māori had an increased risk of an overall unfavorable outcome at 3, 6, and 12 months (adjusted OR = 1.6, 95% confidence interval (CI) = (1.3–1.9); 1.4 (1.2–1.7); and 1.4 (1.2–1.7), respectively; Figure 1). The risk of death for Māori was significantly higher at all time-points (1.7 (1.3–2.1); 1.5 (1.2–1.9); 1.7 (1.3–2.1)). Change in residence was also higher at all time-points and statistically significant at 3 and 6 months (1.6 (1.3–2.1); 1.3 (1.1–1.7); 1.2 (1.0–1.5)). Finally, unemployed had increased at all time-points and statistically significant at 6 and 12 months (1.4 (1.0–2.1); 1.5 (1.1–2.1); 1.5 (1.1–2.1)). We found no associations with Pacific or Asian ethnicity. Excluding people who were not working at the time of stroke did not significantly change the results nor did excluding people with previous stoke (data not shown). Outcomes for people with I63 = cerebral infarction and I64 = stroke unspecified subtypes did not differ to all strokes combined. For the I61 = intracerebral hemorrhage group, unemployed increased at all three time-points for Asians and became significant at 6 and 12 months (2.3 (0.9–6.1); 2.4 (1.0–5.6); 2.3 (1.0–5.1)). For Pacific Peoples with I61, the risk of an overall unfavorable outcome was decreased and statistically significant at 3 and 6 months (0.5 (0.3–0.8); 0.6 (0.3–1.0); 0.6 (0.3–1.1)).

### Thrombectomy and post-stroke medication prescriptions

**Figure 2.**
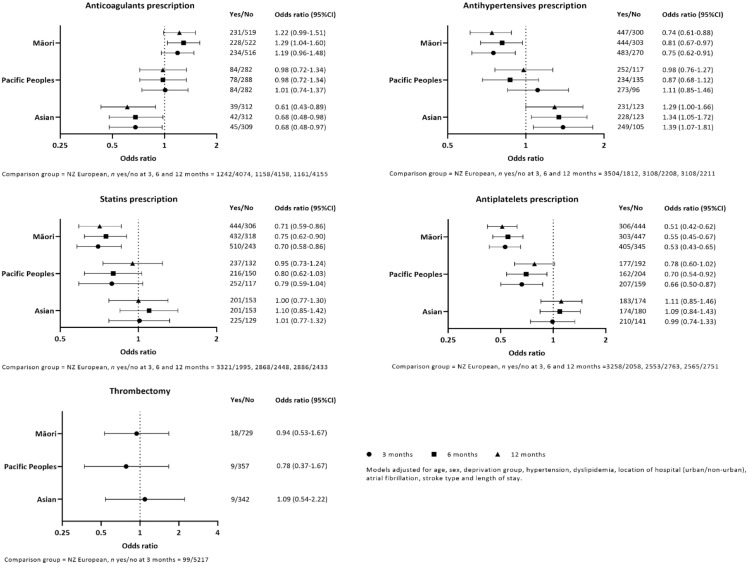
Likelihood of thrombectomy and post-stroke medication prescriptions by ethnicity.

There was no difference in accessing thrombectomy between ethnic groups; however, numbers were small for this outcome. Compared with NZ Europeans, Māori were prescribed fewer antihypertensives, statins, and antiplatelets at all three time-points and more anticoagulants at 6 months. Pacific Peoples were prescribed fewer antiplatelets at 3 and 6 months. There were fewer anticoagulant and more antihypertensives prescriptions for Asians at all time-points (Figure 2).

## Discussion

We found significantly worse post-stroke outcomes for Māori compared with NZ Europeans; disparities among Māori, Pacific, and Asian people in accessing post-stroke secondary prevention medications were also observed.

Greater job loss for Māori could be due to Māori being younger at stroke onset, when they are more likely to be of working age. However, a similar pattern would be expected for Pacific Peoples who also experience stroke at a younger age.

Several risk factors, including smoking and diabetes, were more common among Māori than NZ Europeans, which likely explains some differences, as described previously.^17^ Stroke etiology and type also differed between ethnic groups potentially explaining why Pacific Peoples and Asians had better outcomes than Māori as the higher rate of atrial fibrillation among Māori may have resulted in more severe strokes.^18^ However, the atrial fibrillation rate in NZ Europeans was similar, and Pacific Peoples and Asians experienced more intracerebral hemorrhages, which are typically associated with the most severe strokes.^19^ Furthermore, length of stay, a marker of severity, was similar between groups and controlled for in the analysis along with stroke type, suggesting that these factors are unlikely to fully explain poorer outcomes among Māori. While it was not a main objective, sensitivity analyses suggested there may be some differences in outcomes by stroke subtype, warranting further investigation.

In contrast to this study, previous studies finding ethnic disparities in post-stroke outcomes in NZ, for example, the Auckland Regional Community Stroke Study (ARCOS IV), reported similarities between Pacific Peoples and Māori, and to a lesser degree Asian people.^20^ Data for ARCOS IV were collected between 2011 and 2012, and it is possible that treatments introduced in more recent years have benefited other groups more than Māori. Indeed, we recently reported greater access to secondary prevention support for Pacific Peoples compared with Māori patients.^21^ Also, overall stroke service improvements may have been implemented more rapidly in urban areas (more Pacific Peoples attended urban hospitals compared with Māori (91% vs 51%)), and we have recently shown that patients presenting to non-urban hospitals experience worse outcomes.^22^ However, this cannot fully explain poorer outcomes for Māori as the proportion attending urban area hospitals is similar to that of NZ Europeans (51% and 61%, respectively), and we controlled for hospital location.

There were differences in medication dispensing following discharge. Some of these may relate to differences in stroke type, for example, Māori may require less antihypertensives as their main risk factor is atrial fibrillation and Asians may require fewer antiplatelets as they have more hemorrhagic strokes. However, risk factors and stroke types were controlled for. Disparities in appropriate prescriptions, as demonstrated in related conditions,^23^ raise concerns about health provider choices that are unrelated to underlying pathology and may instead be informed by personal beliefs around anticipated adherence or other unconscious bias. These findings should prompt practitioner self-reflection and service-level initiatives to monitor and address these potential barriers.

We observed no differences in thrombectomy. However, this was based on small numbers; thus, analyses may have been underpowered to detect this.

Socioeconomic factors have been implicated in stroke patterns.^24^ However, hospital attendance is free in NZ, and prescriptions are heavily subsidized. In addition, adjusting for socioeconomic status showed no differences in outcomes, confirming that this was unlikely to be a strong risk factor in this cohort. Nonetheless, socioeconomic status impacts other risk/protective factors such as transport, childcare and leisure time to enable access to primary health care, healthy foods, and physical exercise.

In the absence of a clear explanation of why Māori experience poorer post-stroke outcomes and secondary prevention implementation, it is important to consider that Māori may experience specific ethnicity-based social disadvantages. Health and stroke disparities between indigenous and other ethnic minority peoples have been described internationally,^2,25,26^ and underlying causes, including colonization and institutional racism, are well described.^27–29^ There are widely available cultural support services for Māori at all NZ hospitals; however, these are inconsistently offered by stroke teams.^22^ Enhancing cultural awareness and safety, as well as increasing diversity among the stroke workforce to improve trust and culturally safe care, represent opportunities for improvement.^30^ Recent health system reforms in NZ include the establishment of a Māori Health Authority, Te Aka Whai Ora, which will ensure greater influence of Māori throughout the health system and support self-determination and indigenous innovation. These approaches need to be introduced alongside ongoing efforts to optimize pre-stroke risk factor management in primary care, although, here too, recognition of unconscious bias is important. In addition, the notion that all stroke risk factor patterns are due to “poor” patient behavior among disadvantaged populations needs to be refuted.^31^

There is also a need for stroke researchers to standardize approaches to collecting and reporting data about minority and indigenous populations. Not stratifying by ethnicity could overlook important differences in stroke risk factors, incidence, access to care, and outcomes, with resulting interventions potentially increasing, rather than reducing, health inequity.

The study has several strengths. It is a comprehensive national cohort with sufficient sample size and reliable follow-up allowing differences by ethnicity to be assessed. The surrogate outcome for functional independence provides valuable information about post-stroke status beyond mortality. Information on length of stay, a marker of stroke severity, allowed control for a strong predictor of post-stroke outcome. Finally, access to other governmental data enabled adjustment for employment and social deprivation, addressing further potential confounding.

Limitations include that the IDI does not contain data on key stroke interventions such as thrombolysis or acute stroke unit care; this could therefore not be taken into account but was explored in a smaller study using non-administrative data.^21^ Furthermore, as health administrative data rely on hospital coders for diagnostic assignment, we cannot exclude the possibility that some individuals with stroke were missed or incorrectly assigned a stroke diagnosis. We made assumptions about medication dispensing to identify baseline risk factors that may have resulted in incorrect risk factor identification in some cases (Supplemental Table S2). In addition, while length of stay is associated with stroke severity,^16^ it may be impacted by other factors. However, as these services are provided under a universal health care system, thus reducing inequality, it is unlikely that the use of this marker would have biased the results. Some people may have moved residence after stroke for reasons other than needing additional support with daily activities, leading to misclassification. However, the number is likely to be small, and misclassification is unlikely to be considerably different among ethnic groups. Finally, this study focused on NZ ethnic populations and may not be generalizable to other populations. However, similar themes have been observed elsewhere, and this work adds to the global evidence of disparities among indigenous and ethnic minority populations.

In conclusion, there are significant disparities in post-stroke outcomes for Māori with a suggestion of poorer access to some key post-stroke secondary prevention medications for Māori and other minority populations. These differences were not fully explained by differences in risk factors or socioeconomic deprivation, suggesting that they may be attributable, at least in part, to stroke service delivery rather than patient factors. Reducing these gaps represents a high priority for future stroke service planning, which may require addressing systemic cultural barriers, unconscious bias, improving cultural safety practices, and ongoing monitoring, in addition to efforts to optimize primary prevention risk factor management.

## Supplemental Material

sj-docx-1-wso-10.1177_17474930231164024 – Supplemental material for Ethnic differences in stroke outcomes in Aotearoa New Zealand: A national linkage study Click here for additional data file. Supplemental material, sj-docx-1-wso-10.1177_17474930231164024 for Ethnic differences in stroke outcomes in Aotearoa New Zealand: A national linkage study by Hayley J Denison, Marine Corbin, Jeroen Douwes, Stephanie G Thompson, Matire Harwood, Alan Davis, John N Fink, P Alan Barber, John H Gommans, Dominique A Cadilhac, William Levack, Harry McNaughton, Joosup Kim, Valery L Feigin, Virginia Abernethy, Jackie Girvan, Andrew Wilson and Anna Ranta in International Journal of Stroke

sj-docx-2-wso-10.1177_17474930231164024 – Supplemental material for Ethnic differences in stroke outcomes in Aotearoa New Zealand: A national linkage study Click here for additional data file. Supplemental material, sj-docx-2-wso-10.1177_17474930231164024 for Ethnic differences in stroke outcomes in Aotearoa New Zealand: A national linkage study by Hayley J Denison, Marine Corbin, Jeroen Douwes, Stephanie G Thompson, Matire Harwood, Alan Davis, John N Fink, P Alan Barber, John H Gommans, Dominique A Cadilhac, William Levack, Harry McNaughton, Joosup Kim, Valery L Feigin, Virginia Abernethy, Jackie Girvan, Andrew Wilson and Anna Ranta in International Journal of Stroke

## References

[1] GBD 2016 Stroke Collaborators. Global, regional, and national burden of stroke, 1990-2016: a systematic analysis for the Global Burden of Disease Study 2016. Lancet Neurol 2019; 18: 439–458. 30871944 10.1016/S1474-4422(19)30034-1 PMC6494974

[2] Schwamm LH Reeves MJ Pan W , et al. Race/ethnicity, quality of care, and outcomes in ischemic stroke. Circulation 2010; 121: 1492–1501. 20308617 10.1161/CIRCULATIONAHA.109.881490

[3] Ellis C Hyacinth HI Beckett J , et al. Racial/Ethnic differences in poststroke rehabilitation outcomes. Stroke Res Treat 2014; 2014: 950746. 25028619 10.1155/2014/950746 PMC4084586

[4] Mendy VL Vargas R Payton M Sims JN Zhang L . Trends in the stroke death rate among Mississippi adults, 2000-2016. Prev Chron Dis 2019; 16: E21. 10.5888/pcd16.180425 PMC6395077 30767859

[5] Ideta TR Lim E Nakagawa K Koenig MA . Racial and ethnic disparities in hospital mortality among ischemic stroke patients in Hawaii. J Stroke Cerebrovasc Dis 2018; 27: 1458–1465. 29433932 10.1016/j.jstrokecerebrovasdis.2017.12.042

[6] Schieb LJ Ayala C Valderrama AL Veazie MA . Trends and disparities in stroke mortality by region for American Indians and Alaska Natives. Am J Public Health 2014; 104: S368–S376. 24754653 10.2105/AJPH.2013.301698 PMC4035883

[7] Hanchate AD Schwamm LH Huang W Hylek EM . Comparison of ischemic stroke outcomes and patient and hospital characteristics by race/ethnicity and socioeconomic status. Stroke 2013; 44: 469–476. 23306327 10.1161/STROKEAHA.112.669341 PMC3595403

[8] Cruz-Flores S Rodriguez GJ Chaudhry MRA , et al. Racial/ethnic disparities in hospital utilization in intracerebral hemorrhage. Int J Stroke 2019; 14: 686–695. 30868940 10.1177/1747493019835335

[9] Berges IM Kuo YF Ottenbacher KJ Seale GS Ostir GV . Recovery of functional status after stroke in a tri-ethnic population. PM R 2012; 4: 290–295. 22541375 10.1016/j.pmrj.2012.01.010 PMC3496396

[10] Goldstein LB Matchar DB Hoff-Lindquist J Samsa GP Horner RD . Veterans Administration Acute Stroke (VASt) Study: lack of race/ethnic-based differences in utilization of stroke-related procedures or services. Stroke 2003; 34: 999–1004. 10.1161/01.STR.0000063364.88309.27 12649513

[11] Horner RD Swanson JW Bosworth HB Matchar DB . Effects of race and poverty on the process and outcome of inpatient rehabilitation services among stroke patients. Stroke 2003; 34: 1027–1031. 12624220 10.1161/01.STR.0000060028.60365.5D

[12] Busch MA Coshall C Heuschmann PU McKevitt C Wolfe CD . Sociodemographic differences in return to work after stroke: the South London Stroke Register (SLSR). J Neurol Neurosurg Psychiatry 2009; 80: 888–893. 19276102 10.1136/jnnp.2008.163295

[13] Ranta A Thompson S Harwood MLN , et al. Reducing ethnic and geographic inequities to optimise New Zealand stroke care (REGIONS care): protocol for a nationwide observational study. JMIR Res Protoc 2021; 10: e25374. 10.2196/25374 PMC7838000 33433396

[14] Milne BJ Atkinson J Blakely T Day H . Data resource profile: the New Zealand integrated data infrastructure (IDI). Int J Epidemiol 2019; 48: e677. 10.1093/ije/dyz054 30879058

[15] Salmond C Crampton P Atkinson J . NZDep2006 index of deprivation. Wellington, New Zealand: Department of Public Health, University of Otago Wellington, 2007.

[16] Kang JH Bae HJ Choi YA Lee SH Shin HI . Length of hospital stay after stroke: a Korean nationwide study. Ann Rehabil Med 2016; 40: 675–681. 27606274 10.5535/arm.2016.40.4.675 PMC5012979

[17] Barber PA Krishnamurthi R Parag V , et al. Incidence of transient ischemic attack in Auckland, New Zealand, in 2011 to 2012. Stroke 2016; 47: 2183–2188. 27470991 10.1161/STROKEAHA.116.014010

[18] Gu Y Doughty RN Freedman B , et al. Burden of atrial fibrillation in Maori and Pacific people in New Zealand: a cohort study. Intern Med J 2018; 48: 301–309. 29034985 10.1111/imj.13648

[19] Bhalla A Wang Y Rudd A Wolfe CD . Differences in outcome and predictors between ischemic and intracerebral hemorrhage: the South London Stroke Register. Stroke 2013; 44: 2174–2181. 23813988 10.1161/STROKEAHA.113.001263

[20] Feigin VL Krishnamurthi RV Barker-Collo S , et al. 30-year trends in stroke rates and outcome in Auckland, New Zealand (1981-2012): a multi-ethnic population-based series of studies. PLoS ONE 2015; 10: e0134609. 10.1371/journal.pone.0134609 PMC4546383 26291829

[21] Thompson SG Barber PA Gommans JH , et al. The impact of ethnicity on stroke care access and patient outcomes: a New Zealand nationwide observational study. Lancet Reg Health West Pac 2022; 20: 100358. 35036976 10.1016/j.lanwpc.2021.100358 PMC8743211

[22] Thompson SG Barber PA Gommans JH , et al. Geographic disparities in stroke outcomes and service access: a prospective observational study. Neurology 2022; 1–36. 10.1212/WNL.0000000000200526 PMC9421775 35623890

[23] Chepulis L Mayo C Morison B Keenan R Lao C Paul R Lawrenson R . Metformin adherence in patients with type 2 diabetes and its association with glycated haemoglobin levels. J Prim Health Care 2020; 12: 318–326. 33349319 10.1071/HC20043

[24] Avan A Digaleh H Di Napoli M , et al. Socioeconomic status and stroke incidence, prevalence, mortality, and worldwide burden: an ecological analysis from the Global Burden of Disease Study 2017. BMC Med 2019; 17: 191. 31647003 10.1186/s12916-019-1397-3 PMC6813111

[25] Tiedeman C Suthers B Julien B Hackett A Oakley P . Management of stroke in the Australian Indigenous population: from hospitals to communities. Intern Med J 2019; 49: 962–968. 30907045 10.1111/imj.14303

[26] Coutinho JM Klaver EC Roos YB Stam J Nederkoorn PJ . Ethnicity and thrombolysis in ischemic stroke: a hospital based study in Amsterdam. BMC Neurol 2011; 11: 81. 21714938 10.1186/1471-2377-11-81 PMC3145566

[27] Ellison-Loschmann L Pearce N . Improving access to health care among New Zealand’s Maori population. Am J Public Health 2006; 96: 612–617. 16507721 10.2105/AJPH.2005.070680 PMC1470538

[28] Harris R Tobias M Jeffreys M Waldegrave K Karlsen S Nazroo J . Effects of self-reported racial discrimination and deprivation on Maori health and inequalities in New Zealand: cross-sectional study. Lancet 2006; 367: 2005–2009. 16782491 10.1016/S0140-6736(06)68890-9

[29] Barnes HM McCreanor T . Colonisation, hauora and whenua in Aotearoa. J Roy Soc New Zeal 2019; 49: 19–33.

[30] Jansen P Bacal K Crengle S . He Ritenga Whakaaro: Māori experiences of health services. Auckland, New Zealand: Mauri Ora Associates, 2008.

[31] Levine DA Duncan PW Nguyen-Huynh MN Ogedegbe OG . Interventions targeting racial/ethnic disparities in stroke prevention and treatment. Stroke 2020; 51: 3425–3432. 33104466 10.1161/STROKEAHA.120.030427 PMC7594115

