# Supplementary material for: Ethnic differences in stroke outcomes in Aotearoa New Zealand: A national linkage study
Source: Int J Stroke. 2023 Mar 24;18(6):663–71. doi: 10.1177/17474930231164024 (PMC10311930; doi:10.1177/17474930231164024)
Supplement: sj-docx-1-wso-10.1177_17474930231164024 – Supplemental material for Ethnic differences in stroke outcomes in Aotearoa New Zealand: A national linkage study [file sj-docx-1-wso-10.1177_17474930231164024.docx]

| **Table S2. Definitions of comorbidities included as covariates in analysis models** | |
| --- | --- |
| **Comorbidity** | **Definition** |
| Hypertension | Two or more pharmaceutical dispensings of anti-hypertensive medication during the ten-year period prior to stroke, or a record of a public hospital discharge with a high blood pressure related ICD code (ICD-9-AM 401.0, 401.1, 401.9, 402, 403/4 and ICD-10-AM I10, I11, I12/3) |
| Dyslipidemia | Two or more pharmaceutical dispensings of medication used to lower cholesterol during the ten-year period before stroke or a public hospital discharge with a high cholesterol related ICD code (ICD-9-AM 272.0, 272.4 and ICD-10-AM E780, E785) |
| Atrial fibrillation | Two or more pharmaceutical dispensings of anticoagulant medication during the ten-year period before stroke or a public hospital discharge with one of the following ICD codes (ICD-9-AM 427.3 and ICD-10-AM I48) |
