# Supplementary material for: Ethnic differences in stroke outcomes in Aotearoa New Zealand: A national linkage study
Source: Int J Stroke. 2023 Mar 24;18(6):663–71. doi: 10.1177/17474930231164024 (PMC10311930; doi:10.1177/17474930231164024)
Supplement: sj-docx-2-wso-10.1177_17474930231164024 – Supplemental material for Ethnic differences in stroke outcomes in Aotearoa New Zealand: A national linkage study [file sj-docx-2-wso-10.1177_17474930231164024.docx]

| **Table S2. Detailed frequencies for hypertension, hyperlipidaemia, and atrial fibrillation** |  | |  | |  | |  | |
| --- | --- | --- | --- | --- | --- | --- | --- | --- |
|  | **NZ European** | | **Māori** | | **Pacific Peoples** | | **Asian** | |
|  | n | % | n | % | n | % | n | % |
| **Total** | 5394 |  | 762 |  | 369 |  | 354 |  |
| **Hypertension** |  |  |  |  |  |  |  |  |
| Blood pressure lowering medication^a^ (I) | 4281 | 79.4% | 588 | 77.2% | 309 | 83.7% | 255 | 72.0% |
| Hypertension diagnosis in hospital (II) | 2427 | 45.0% | 357 | 46.9% | 189 | 51.2% | 138 | 39.0% |
| Hypertension composite variable (I or II) | 4341 | 80.5% | 597 | 78.3% | 312 | 84.6% | 258 | 72.9% |
| On blood pressure lowering medication at the time of stroke^b^ | 3669 | 68.0% | 486 | 63.8% | 249 | 67.5% | 219 | 61.9% |
| **Dyslipidemia** |  |  |  |  |  |  |  |  |
| Cholesterol lowering medication^a^ (III) | 3243 | 60.1% | 462 | 60.6% | 246 | 66.7% | 189 | 53.4% |
| Hyperlipidemia diagnosis in hospital (IV) | 744 | 13.8% | 105 | 13.8% | 72 | 19.5% | 51 | 14.4% |
| Hyperlipidemia composite variable (III or IV) | 3285 | 60.9% | 468 | 61.4% | 249 | 67.5% | 192 | 54.2% |
| On cholesterol lowering medication at the time of stroke^b^ | 2169 | 40.2% | 327 | 42.9% | 180 | 48.8% | 141 | 39.8% |
| **Atrial fibrillation** |  |  |  |  |  |  |  |  |
| Anticoagulant medication^a^ (V) | 1254 | 23.2% | 234 | 30.7% | 72 | 19.5% | 36 | 10.2% |
| Anticoagulant medication^a^ excluding people with pulmonary embolism or DVT diagnoses | 1113 | 20.6% | 216 | 28.3% | 66 | 17.9% | 30 | 8.5% |
| Anticoagulant medication for more than one year (in 10 years prior to stroke) | 954 | 17.7% | 189 | 24.8% | 60 | 16.3% | 21 | 5.9% |
| Atrial fibrillation diagnosis in hospital (VI) | 1320 | 24.5% | 222 | 29.1% | 72 | 19.5% | 36 | 10.2% |
| On anticoagulant medication at the time of stroke^b^ | 924 | 17.1% | 183 | 24.0% | 57 | 15.4% | 27 | 7.6% |
| Atrial fibrillation (composite variable) (V or VI) | 1722 | 31.9% | 285 | 37.4% | 93 | 25.2% | 54 | 15.3% |

^a^Two or more prescriptions in the 10 years prior to stroke event

^b^One or more prescriptions in the 6 months prior to stroke event
